# Association between rs3761548 polymorphism of *FOXP3* and the risk of gastric cancer: a case-control study

**DOI:** 10.22099/mbrc.2024.49125.1989

**Published:** 2024

**Authors:** Shamimeh Validi, Iraj Saadat

**Affiliations:** Department of Biology, Faculty of Science, Shiraz University, Shiraz, Iran

**Keywords:** FOXP3, Gastric Cancer, Genetic Polymorphisms, PCR-RFLP

## Abstract

Gastric cancer is one of the most prevalent malignancies in the world. Various factors play a role in the development of this disease as risk factors. One of these genes is the *FOXP3*, which is located on the short arm of the X chromosome (Xp11.23). The rs3761548 polymorphism in the promoter region of this gene increases cell proliferation. In the current study, the association between this genetic polymorphism and the risk of gastric cancer was investigated. This study included 147 patients (55 women, 92 men) with gastric cancer and 147 healthy individuals (53 women, 94 men). The PCR-RFLP method is used for genotyping. Statistical analysis showed that there was no significant association between this polymorphism and the risk of gastric cancer. However, the analysis of genotype, family history and smoking risk factors simultane-oussly revealed a significant relationship between simultaneous occurrence of two (OR=3.79, 95% CI=1.77-8.09, p=0.001) and three risk factors (OR=6.44, 95% CI=1.76-23.5, p=0.017) and the risk of gastric cancer.

## INTRODUCTION

Gastric cancer is the fourth most common malignancy worldwide and the second leading cause of death among all malignancies in the world, ranking fourth among men and fifth among women [1]. The development of gastric cancer is a multifactorial process and many conditions increase its risk. Family history of gastric cancer, *Helicobacter pylori* infection (a common bacterium that can cause gastric ulcers), a history of gastric adenomatosis more than 2 cm, a history of chronic gastric atrophy, a history of severe anemia, obesity, alcoholism, excessive consumption of red meat and low economic status can be important in inducing this cancer   [2] . Recent research suggests that mononucleotide polymorphisms may shed light on tumorigenesis in gastric cancer. Polymorphisms in various genes such as *MMP-9*, *E-cadherin*, *EGF, HER-2*, and* MMP-1* can increase the risk of gastric cancer [3, 4]. 

Regulatory T (Treg) cells play an important role in the immune suppressive system and are subgroups of CD4. FOXP3 (MIM: 300292) as a transcription factor is a marker molecule required for Treg cells [5], specifically through IL2R, TGFβ, STAT, SMAD, PI3K signaling pathways, can express the transcription factor FOXP3, which is a subset of the forkhead family [6, 7]. This transcription factor can play an important role in the differentiation and proliferation of regulatory T cells [8, 9]. 

The *FOXP3* gene has 14392 nucleotides with 11 exons located on the short arm of the X chromosome (Xp11.23). The total length of the human FOXP3 protein is 431 amino acids with a molecular weight of 47.24 kDa [10]  . Significantly, a large number of Treg cells have been found in the blood of cancer patients, and as a result of the activity of these cells, the antitumor response has also decreased [11]. Due to this, the percentage of Treg cells at the systematic level shows a close relationship with the prognosis and survival of gastric cancer [12]. Therefore, it can be said that increasing the expression of the *FOXP3* can increase the number of Treg cells, which in turn suppresses the immune response against the tumor, which results in the progression of cancer [13]. 

Several single nucleotide polymorphisms were found in the intron, exon, and promoter regions of the *FOXP3* [14]. It was thought that polymorphism, which is present in the promoter region of the *FOXP3*, may affect the function or quantity of Treg, which in turn leads to an immune diversion against the tumor [15, 16]. Single nucleotide polymorphism rs3761548 is one of the polymorphisms in the promoter region and serves as a binding site for Sp1 (Specificity protein 1) transcription factor. Hence, this polymorphism can affect the interaction between the *FOXP3* promoter and Sp1 [17]. 

An association between the rs3761548 and gastric cancer has been shown in a recent study from the population of Tabriz (Northwest Iran) [18]. Iranian population is one of the most heterogeneous populations [19], so we collected samples from people who live in Fars province (Southwest Iran). Identifying different genetic variants is very useful for the prediction, prevention and early treatment of many diseases, so in the present study we examined the association between rs3761548 and the risk of gastric cancer. 

## MATERIALS AND METHODS

**Study Subjects: **A total of 147 gastric cancer pateints (selecting from the chemotherapy department of Namazi Hospital) and 147 healthy individuals (selecting from the healthy blood donor), who matched for age and gender, participated in our case-control study. The mean age in healthy controls and pateints was 54.7 ± 8.3 and 57.2 ± 13.3 years, respectively. There was no significant difference in age between the two groups (p=0.59). The two groups were also sex-matched with each other (p=0.809). We chose our case and control group from Iranian Muslims living in Fars province. Prior to the study, consent was obtained from the participants. Each individual was asked to complete a self-test questionnaire by providing demographic information, smoking status, and family history of first-degree relatives with cancer. Anyone with at least one first-degree relative with cancer considered a case with a positive family history. 

**Genotyping analysis: **The required genomic DNA was extracted from whole blood samples according to standard protocole and stored at -20°C in frezzer until use [20]. Genotyping of *FOXP3* polymorphism was performed by PCR-RFLP method. In order to amplify the polymorphic site, primers 5′-TAACCAGACAGCGTAGAAGG-3′ (forward) and 5′-CAATACAGAGCCCATCATCA-3′ (reverse) were used [18].

**Figure 1:**
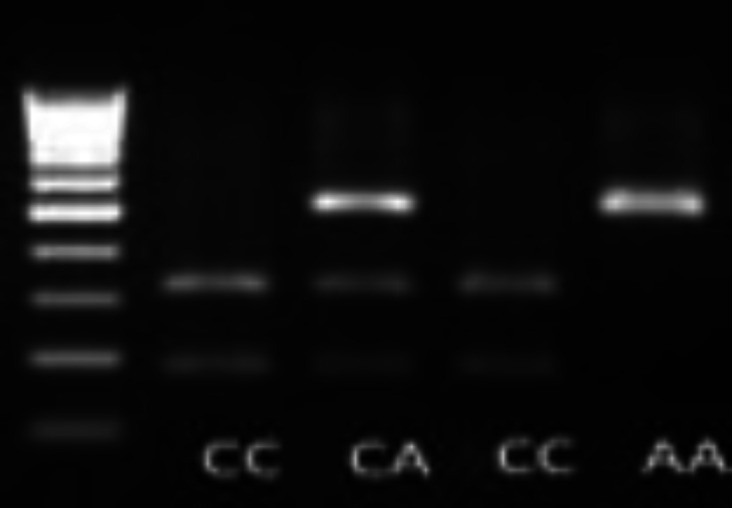
Genotyping of *FOXP3* polymorphism (rs3761548) by PCR-RFLP technique. Lane 1, 100 bp ladder.

The PCR conditions consisted of an initial denaturation step of 94°C for 5 min, followed by 32 cycles of 94°C for 30 s, 60°C for 30 s and 72°C for 30 s, a final extension of 72°C for 5 min. The amplified PCR product includes the rs3761548 with the length of 503 bp. For enzymatic digestion of the desired polymorphism, *Pst I* enzyme was used. The fragment length of allele A is 503 bp, and for allele C is 319 and 184 bp (Fig. 1).

**Statistical analysis: **Hardy-Weinberg equilibrium (HWE) was assessed using the chi-squared test, as recommended elsewhere [21, 22]. In order to evaluate the association between genotype and the risk of gastric cancer, we used odds ratio (OR) and 95% confidence interval (CI). Statistical analyses for the polymorphic X-linked loci were performed as recommended elsewhere [23]. All data were analyzed with SPSS software version 24. A probability of p<0.05 was considered to be statistically significant.

## RESULTS

General characteristics of information based on gastric cancer and control groups were shown in Table 1. The difference between control and patient in terms of smoking was statistically significant (OR=2.25, 95% CI=1.34-3.77, p=0.002). Family history, which indicates the genetic background of the individual, also with OR=2.61, 95% CI=1.42-4.78, p=0.002, showed that it is a risk factor for gastric cancer (Table 1). 

**Table 1:** Selected characteristics of participants of gastric cancer study

**Characteristics**	**Controls (n=147)**	**Cases (n=147)**	**OR**	**95% CI**	**p-value**
**Gender**					
Female, N (%)	53	55	-	-	0.809
Male, N (%)	94	92			
**Tobacco smoking **					
Non-smokers	112	85	1.0	-	-
Smoking	34	56	2.25	1.349-3.772	0.002
Missing	1	6	-	-	-
**Family history**					
Negative	128	40	1.0	-	-
Positive	19	103	2.61	1.429-4.789	0.002
Missing	-	4	-	-	-

CI = confidence interval, OR = odds ratio, P<0.05

The Comparison of genotypic and allelic frequencies of *FOXP3* polymorphism in control and patient groups was shown in Table 2. The frequency of C and A alleles in the control group was 0.590 and 0.410, respectively. There was no significant deviation between observed and expected values based on HWE for the genotypes ($x^{2}$=3.34, df=1, p>0.05).

In this analysis we chose the C allele in men as a reference and measured the A allele frequency relative to it. According to the results, there was no significant relationship between the indication of A allele and the risk of gastric cancer (OR=0.69, CI=0.61-1.97, p=0.65). In females, we also chose the CC genotype as a reference and examined AC and AA genotypes based on it. The results showed that there was no significant relationship between AA and AC genotypes with gastric cancer (AA vs CC: OR=0.70, 95% CI=0.30-1.63, p=0.411) (AC vs CC: OR= 0.405, 95% CI=0.13-1.25, p=0.117).

In next step, we also evaluated the additive effect of three risk factors (genotype, smoking, family history) on gastric cancer. Table 3 shows the number of participants who have zero to three risk factors. 

**Table 2:** Distributions of polymorphisms *FOXP3* rs3761548 in gastric cancer cases and controls

**Sex**	**Genotypes**	**Cases **	**Controls **	**OR**	**95% CI**	**P-value**
**Females**	**CC**	31	23	1.00	-	-
	**AC**	18	19	0.405	0.13-1.25	0.117
	**AA**	6	11	0.703	0.30-1.63	0.411
**Males**	**C**	54	53	1.00	-	-
	**A**	38	41	0.964	0.61-1.97	0.651

CI=confidence interval, OR=odds ratio, P<0.05

**Table 3:** The number of people who have zero to three risk factors

**Genotype**	**History**	**Cigarettes**	**Control**	**Case**	**Risk Factor**
AA	-	-	7	2	0
AA	-	+	2	1	1
AA	+	-	2	2	1
AA	+	+	-	-	2
AC/CC	-	-	24	26	1
AC/CC	-	+	8	4	2
AC/CC	+	-	6	14	2
AC/CC	+	+	3	4	3
A	-	-	30	14	0
A	-	+	8	17	1
A	+	-	2	3	1
A	+	+	1	3	2
C	-	-	38	15	1
C	-	+	10	19	2
C	+	-	4	7	2
C	+	+	1	7	3

Table 4 shows the additive effect of three risk factors for gastric cancer development. Risk factor 0 means (there is no risk factor), 1 means (existence of one risk factor), 2 means (existence of two risk factors) and 3 means (existence of three risk factors). For multivariable analysis, variables with p<0.20 in univariable analysis were used. In our study, the OR of AC/CC genotype compared with AA genotype is greater than 1 with P value of 0.10. Therefore, we included the genotypes as well as smoking habit and family history in the multivariable analysis. Our results showed that there is a strong correlation between the number of risk factors and the incidence of gastric cancer in individuals. Individuals with a single risk factor, the risk of cancer increases by about 1.9 times (OR=1.96, 95% CI= 0.99-3.90, p=0.053); individuals with two risk factors, the risk increases by about 3.8 times (OR=3.79, 95% CI=1.77-8.09, p=0.001), and in people with three risk factors, the risk increases by about 6.4 times (OR=6.44, 95% CI=1.76-23.5, p=0.017).

**Table 4:** Additive effect of genotype, smoking, and family history on the risk of gastric cancer

**Number of risk factors**	**OR**	**%95 CI**	**p-value**
0	1	-	-
1	1.969	0.993-3.906	0.053
2	3.792	1.777-8.095	0.001
3	6.449	1.766-23.547	0.017

CI = confidence interval, OR = odds ratio, P<0.05

## DISCUSSION

Several factors play a role in the development of gastric cancer and several genes play a role in this disease. One of these genes is the *FOXP3* which has been defined as a transcription factor essential for the induction of immunosuppressive functions in regulatory T cells. An in-depth study of the role and underlying mechanism of *FOXP3* in gastric cancer cells is of great importance. This gene usually inhibits the growth of cancer cells by suppressing oncogenes (*HER2*,* c-Myc*, *Skp2*) and increasing the *P21* tumor suppressor gene [24]. 

To date, the association of rs3761548 polymorphism with gastric cancer risk has been reported in only one study [18]. To draw accurate conclusions about genetic polymorphisms and the risk of multifactorial diseases such as cancer, a large number of independent studies should be conducted in different populations. In the present study, after examining the relationship between *FOXP3* genotypes and the risk of gastric cancer, no significant relationship was observed between any of the rs3761548 and the risk of gastric cancer despite previous report [18]. Family history and smoking are important risk factors for gastric cancer. The present study indicated that family history and smoking are associated with the risk of gastric cancer. The simultaneous effect of three risk factors of genotype, smoking and family history as risk factors showed that the simultaneous presence of two and three risk factors significantly increases the risk of gastric cancer (Table 4). If two risk factors are involved, the risk increases by about 3.8 times and if three risk factors are involved, the risk increases by nearly 6.5 times. Previous studies related to stomach cancer show that the number of risk factors associated with increased cancer risk [25]. Based on these findings, it can be concluded that the more these risk factors for gastric cancer increase, the more the occurrence of this cancer increases. 

There are two studies investigating the association between the *FOXP3* (rs3761548) genetic polymorphism and the risk of gastric cancer [present study, 18], with inconsistent results. Therefore, further studies with larger numbers of samples in other populations are suggested to draw general conclusions.
